# CT and MR Imaging in the Local Staging of Primary Malignant Musculoskeletal Neoplasms: Report of the Radiology Diagnostic Oncology Group. *Radiology* 1997; 202:237–246

**DOI:** 10.1080/13577149878181

**Published:** 1998-03

**Authors:** Lynne S. Steinbach

**Affiliations:** University of California Box 0628 San Francisco CA 94143-0628 USA


*Sarcoma* (1998) **2**, 57–58

COMMENT

### CT and MR imaging in the local staging of primary malignant musculoskeletal neoplasms: Report of the radiology diagnostic oncology group. *Radiology* 1997; 202:237–246

LYNNE S. STEINBACH

*University of California, San Francisco, USA*

In 1987, the National Institutes of Health formed the Radiology Diagnostic Oncology Group (RDOG) to perform multi-institutional comparative studies of relevant imaging modalities in the staging of various malignancies. One such study was recently completed: CT and MR imaging in the local staging of primary malignant musculoskeletal neoplasms: Report of the Radiology Diagnostic Oncology Group. *Radiology* 1997; 202:237–46.

The primary investigator is David Panicek, MD, an associate professor of radiology at Memorial Sloan-Kettering Cancer Center. This was a collaborative effort between radiologists and pathologists from the following medical centers: Memorial Sloan-Kettering Cancer Center, Massachusetts General Hospital, University of California Los Angeles and Stanford University Hospital. The study enrolled 367 eligible patients ranging in age from 6 to 89 years with malignant bone or soft tissue neoplasms. Of these, 316 patients were able to complete the study and have their images analyzed. Primary bone tumors were present in 183 patients and primary soft tissue tumors in 133 patients.

This carefully conducted investigation utilized a paired study design in which each patient underwent imaging with computed tomography (CT) and magnetic resonance imaging (MRI) for staging of primary malignant musculoskeletal tumors within a period of 4 weeks prior to surgical resection. For each patient, CT scans were interpreted independently by two radiologists and MR images by two other radiologists at the enrolling institution. The CT and MR images were then interpreted together by two of those radiologists and subsequently reread at the other institutions. Imaging and histopathologic findings were compared and were supplemented when needed with surgical findings. Surgeons were not blinded to diagnosis or imaging studies prior to surgery. Receiver operating characteristic curve analysis and descriptive statistical analysis were performed.

The study concluded that CT and MR imaging were equally accurate in the local staging of malignant bone and soft tissue neoplasms. Combined interpretation of CT and MR images did not statistically significantly improve accuracy. Inter-reader variability was similar for both modalities.

The authors are to be applauded for their efforts. Significant time and energy went into preparation and evaluation of these cases. Some of the conclusions were surprising. It was of interest to discover that the length of the intramedullary tumor and the maximum dimension of the tumor in the soft tissues tended to be overestimated with both CT and MRI. As mentioned in the paper, the soft tissue discrepancy may be related to changes in dimension when measured outside the body following resection.

This study has been a subject of controversy since its publication. Many radiologists believe that MRI has higher soft tissue contrast and multi-planar capability that improves and facilitates evaluation of extent of musculoskeletal tumors. As stated by the authors of this article: "it is possible that other important but less easily quantifiable information is gleaned by the surgeon from MR images and MR increases the surgeon's confidence in the pre-operative staging data". Most radiologists would agree with this.

It is important to look at some of the flaws of the study. One must look at the time-frame of the study and the equipment and methodology used. This study was conducted between 1991 and early 1995. The equipment was state of the art for its time: however, newer CT and MR equipment and software is now available producing the potential to alter the results of the study. Patients who underwent CT had the benefit of additional intravenous

Correspondence to: Lynne S. Steinbach, University of California, Box 0628 San Francisco, CA 94143-0628, USA. Tel: + 1 415 476 1451; Fax: + 1 415 476 8550; E-mail: lynne.steinbach@radiology.ucsf.edu.

1357-714X/98/040057–02 ©1998 Carfax Publishing Ltd contrast material, whereas those who had MRI did not receive intravenous contrast, which is often administered by radiologists for improved characterization and visualization of musculoskeletal tumors, particularly to evaluate the soft tissue component. Spin-echo MR imaging techniques were utilized. Short tau inversion recovery (STIR) sequences, and the newer fat-suppressed fast spin echo T2-weighted MR sequences, which are very sensitive for tumors, were not employed. This discrepancy in methodology could bias the results towards CT.

Another important fact that should be mentioned is that the study was skewed predominantly towards evaluation of the more common bone and soft tissue tumors—osteosarcoma, chondrosarcoma, malignant fibrous histiocytoma and liposarcoma. Round cell tumors were excluded by design because they respond to pre-operative therapy and there is little mass left to evaluate at surgery and pathology.

A relatively small number of patients (15) had neurovascular involvement, which weakens statistical power for conclusions that MR and CT are limited in their ability to assess neurovascular encasement. However, the fact that neurovascular involvement occurred rarely is important information in and of itself.

It is recommended that the decision to use of CT or MRI be applied on an individual basis. It will always be difficult to keep up with technological advances in cross-sectional imaging: however, a follow-up study that utilizes state of the art imaging is probably needed to confirm the conclusions of this study.

